# The effect of high-intensity intermittent and moderate-intensity continuous exercises on neurobiological markers and cognitive performance

**DOI:** 10.1186/s13102-024-00831-7

**Published:** 2024-02-07

**Authors:** Yusuf Buzdagli, Murat Ozan, Nurcan Baygutalp, Furkan Oget, Raci Karayigit, Neslihan Yuce, Emirhan Kan, Fatih Baygutalp, Halil Ucar, Yusuf Buzdağlı

**Affiliations:** 1 Department of Coaching Education, Faculty of Sport Sciences, Erzurum Technical University, Erzurum, Turkey; 2 Department of Physical Education and Sport, Kazım Karabekir Faculty of Education, Ataturk University, Erzurum, Turkey; 3 Department of Biochemistry, Faculty of Pharmacy, Ataturk University, Erzurum, Turkey; 4 Department of Physical Education and Sports, Faculty of Sport Sciences, Erzurum Technical University, Erzurum, Turkey; 5 Department of Coaching Education, Faculty of Sport Sciences, Ankara University, Ankara, Turkey; 6 Department of Medical Biochemistry, Faculty of Medicine, Ataturk University, Erzurum, Turkey; 7 Department of Physical Medicine and Rehabilitation, Faculty of Medicine, Ataturk University, Erzurum, Turkey; 8 Winter Sports and Sports Sciences Institute, Ataturk University, Erzurum, Turkey; 9 Faculty of Sports Sciences, Erzurum Technical University, Floor1 Room No:140, Yakutiye, Postal Code: 25500 Erzurum, Turkey

**Keywords:** Exercise, Cognition, Neurobiological, BDNF

## Abstract

**Background:**

The effects of exercise on cognitive functions and general brain health have been increasingly studied. Such studies conducted among athletes are very important to understanding the effects of different exercise methods on biochemical parameters and cognitive performance. The present study aimed to compare the neuroprotective effects of high-intensity interval exercise (HIIE) and moderate-intensity continuous exercise (MICE) based on biochemical parameters and cognitive performance in athletes.

**Methods:**

A total of twenty-eight elite male boxing athletes aged > 18 years, with at least eight years of training experience, who successfully achieved national and international levels were included in this study. The elite athletes participating in the study were aged 24.43 ± 4.72 years, 14.45 ± 5.89 years of training experience, had a body weight of 74.64 ± 7.82 kg, and had a height of 177 ± 7.15 cm. Athletes who consumed any stimulants during the testing or supplementation phase, nutritional supplements, or steroids that may have affected hormone levels or sports performance in the last three months were excluded from this study. Venous blood samples were obtained, and cognitive performance tests (Stroop tests) were applied (i) immediately after high-intensity intermittent exercise (HIIE), (ii) one hour after HIIE, (iii) immediately after moderate-intensity continuous exercise (MICE), and (iv) one hour after MICE. Serum BDNF, S100B, and NSE levels were measured after each session.

**Results:**

Serum BDNF levels were significantly (F = _2.142_, *P* < 0.001, η_p_^2^ = 0.589) greater in the HIIE group (5.65 ± 1.79 ng/mL) than in the control group (1.24 ± 0.54 ng/mL) and MICE group (3.38 ± 1.29 ng/mL) for the samples obtained immediately after exercise. Serum S100B levels were significantly (F = _3.427_, *P* < 0.001, η_p_^2^ = 0.427) greater in the HIIE group (71.92 ± 23.05 ng/L) than in the control group (47.39 ± 15.78 ng/L), however there was no significant difference between the HIIE and MICE groups (59.62 ± 28.90 ng/L) in the samples obtained immediately after exercise. Serum NSE levels were significantly (F = _1.475_, *P* < 0.001, η_p_^2^ = 0.312) greater in the HIIE group (14.57 ± 2.52 ng/mL) than in the control group (9.51 ± 3.44 ng/ML mL), however there was no significant difference between the HIIE and MICE groups (59.62 ± 28.90 ng/L) in the samples obtained immediately after exercise. Compared with control groups, both HIIE and MICE improved cognitive performance demonstrated by the Stroop test results. Again, HIIE was superior to MICE in terms of Stroop task reaction time and error rate (incongruent task) scores.

**Conclusion:**

HIIE and MICE have favorable effects on improving cognitive performance and neuroprotection in an athlete population. HIIE is considered to be superior to MICE in improving neuroprotection and cognitive performance. Our study has remarkable results demonstrating the benefits of HIIT on neuroprotection and cognitive performance. HIIE is recommended instead of MICE, especially in sports where cognitive performance is more important.

## Introduction

 The effect of exercise on cognitive performance has been widely studied since the beginning of the 20th century. Exercise is an important environmental factor that has positive effects on the brain and healthy behavior [1]. More than 60% of the world’s population is insensitive to exercise. Today, it is associated with a sedentary lifestyle and low participation in exercise [2]. Exercise is accepted as a nonpharmacological strategy that has direct effects on functional and cognitive brain structures [3–6]. Many studies have addressed questions about the impact of exercise on cognitive function from two perspectives: chronic ( e.g., weeks, months, and/or years) and acute ( e.g., single session) effects. The current study focuses on the second perspective, which considers the impact of acute exercise on biochemical and cognitive processes.

Many physiological systems contribute holistically exercise at the highest level. The central nervous system, especially the brain, is the command center of these physiological systems. The number and content of studies related to the brain are increasing and deepening day by day. Research on the brain, especially in the field of neuro-exercise under different conditions, has focused on brain responses during and after physical and cognitive loads [7, 8]. Exercise, which is a physiological stress, necessarily causes changes in brain tissue. During and after exercise, biochemical changes occur to ensure the organism’s integrity. The brain undergoes marked changes as a result of stress and exercise [6, 9]. However, the induction of stress at various levels of exercise intensity leads to the examination of more intriguing physiological reactions in the organism [10, 11]. In the current study, using two different exercise models high-intensity interval exercise (HIIE) and moderate-intensity continuous exercise (MICE), stress of different intensities was created, and changes in biochemical and cognitive performance after exercise were evaluated.

Brain-derived neurotrophic factor (BDNF) is a member of the neurotrophic family and plays an essential role in neurodegeneration and neuroprotection [12]. BDNF modulates several brain functions, such as memory and learning, by playing a major role in the development of brain circuits [13]. Previous studies have shown that basal BDNF levels are lower in sedentary individuals than in exercising individuals [14–17]. Moreover, serum BDNF levels were reportedly greater in combat sports athletes after exercise than in athletes in other sports [18]. Studies reporting that exercise has cognitive benefits suggest that BDNF is involved in this mechanism [19, 20]. Exercise type and duration play a role in the effects of exercise on cognitive functions. The proteins S100A8, S100A9, and the heterodimer S100A8/A9, also called calprotectin, are part of the S100 calcium-binding protein family. S100B is a calcium-binding peptide produced by reactive astrocytes and is observed mainly in the cytoplasm of astrocytes [21]. In one study of collegiate football players, plasma levels of S100B were greater after practice than before practice, particularly for players sustaining greater mechanical loading of the head [22]. Another study of football players showed that acute, postgame increases in S100B were significantly associated with greater impact exposure [23]. However, serum S100B expression does not appear to increase reliably after mechanical loading of the head in asymptomatic athletes [24].

Neuron-specific enolase (NSE), a dimeric isoenzyme of the glycolytic enzyme enolase, is found in the cytoplasm of neurons and cells undergoing neuroendocrine differentiation [25]. It is characterized by a relatively high incidence of mild traumatic brain injury (MTBI)/concussion in contact sports [26]. Acute exercise-related changes in the serum concentrations of S-100B and NSE have been shown to be sensitive markers of brain tissue damage [27]. Both of these markers have been reported to affect on long-term neurocognitive abnormalities [28] and cognitive performance [29].

In general, acute exercise is thought to have an inverted-U effect on cognitive performance [30]. The inverted-U hypothesis, which assumes a decrease in cognitive performance at high exercise intensities, has led to many studies [31]. While this theory establishes the general notion that high-intensity exercise has deleterious effects on cognition, experimental studies have consistently failed to detect a clear association [32]. Although there is evidence supporting the Inverted-U theory, it is argued that this effect will not always be observed in athletic populations or individuals with higher fitness levels [10, 33]. In recent years, when studies on the acute effects of exercise on cognitive functions and biochemical changes have been conducted, there has been is no complete consensus.

In this context, a hypothesis was created to evaluate our main hypothesis to determine whether HIIT and MICE exercise regimens have an effect on biochemical changes and cognitive performance in elite boxing athletes. As a result, although it has been suggested that exercise affects biochemical changes and cognitive performance, no comprehensive study has been conducted that quantitatively evaluates the intensity of exercise for these effects. This study examined the effects of exercise applied at different intensities on (a) biochemical changes and (b) cognitive performance.

## Methods

This was a cross-sectional study. This study was approved by the institutional ethics committee of Atatürk University Faculty of Medicine (B.30.2ATA.0.01.00/43) and was conducted at Atatürk University Athlete Performance Measurement Center in accordance with the current version of the Declaration of Helsinki. The high-quality reporting of this cross-sectional study was reported in accordance with the Strengthening the Reporting of Observational Studies in Epidemiology (STROBE) guidelines (www.strobe-statement.org).

### Participants

Twenty-eight elite male boxer athletes participated in this study. The inclusion criteria were as follows: (a) were older than eighteen years, (b) had at least eight years of boxing experience, (c) had a body mass index ≤ 30 kg/m^2^, (d) were male elite athletes, and (e) had obtained national and international degrees. The exclusion criteria were (a) being under eighteen years of age, (b) using stimulants, narcotics, and/or psychoactive substances during the test or supplementing phase, (c) consuming substances such as nutritional supplements or steroids in the past three months that may affect hormone levels or sports performance, and (d) having a history of any orthopedic, neurological, cardiovascular, pulmonary, or metabolic disorder that may adversely affect performance on physical and cognitive tests. Participants were provided information about the research procedure, schedule, and categories of exercises and assessments they needed to complete before signing the informed consent form.

### Study design

**Fig. 1:**
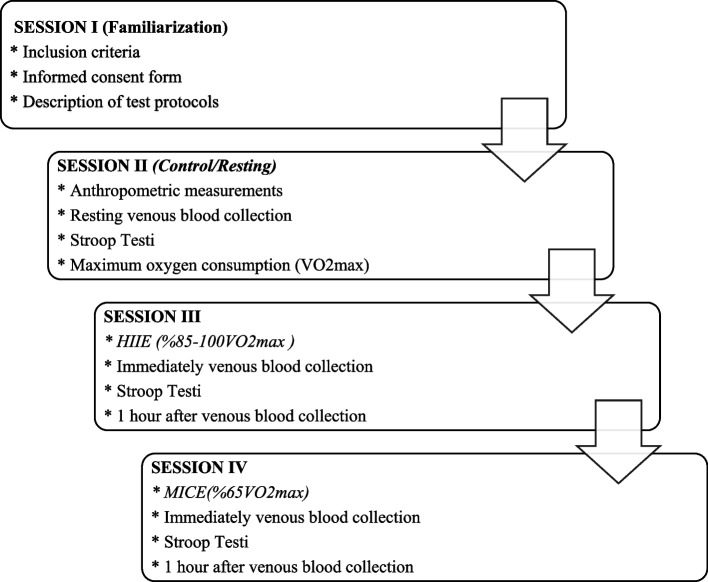
Application stages of test protocols and session demonstrations

 After the necessary information was given to the participants, the familiarization session was applied for a clearer understanding of the exercise protocols. Participants included in the study visited the laboratory four times in total. Anthropometric measurements, maximal oxygen consumption tests, and resting venous blood (2nd session) were taken from the participants after 1 h of fixed rest without exercise in the control session. Afterward, the resting cognitive performance test was administered to the participants. In the second session, venous blood was drawn immediately after high-intensity intermittent exercise was applied, and a cognitive performance test was applied. One hour after HIIE, venous blood was drawn from the participants again. In the third session, venous blood was drawn immediately after moderate-intensity continuous exercise, and a cognitive performance test was applied. One hour after MICE, venous blood was drawn from the participants again (Fig. 1).

### Exercise protocols

The participants’ height, weight, and maximal oxygen consumption test results were recorded at their initial visit to the laboratory for physical and physiological evaluation. Using their credentials and expressions, the researchers determined the ages and sports ages of the athletes participating in the study. A portable height meter was used to assess the height of the athletes (Seca 216, Seca GmbH & Co., Hamburg, Germany). A body composition analyzer (Tanita model TBF-300) was used to measure body composition (Tanita Corp., Tokyo, Japan). In this study, two different running exercise models were applied to examine the effects of acute exercise on biochemical changes and cognitive performance. This exercise model was applied as a continuous (MICE) and interval (HIIE) cycle. These test protocols were performed on a cycle ergometer (Cosmed K5, Italy). Before starting the training sessions, the training intensity of the VO_2_max values determined in the second session, MICE (%65 VO_2_max), and HIIE model (%85–100 VO_2_max) were determined. Exercise protocols were applied on different days and at the same time of day to minimize circadian rhythm effects.

#### High-intensity interval exercise

A standard warm-up of 10 min and an active rest of 3 min were given before each exercise session. Afterward, the exercise protocol was started when the participants were ready. The HIIE protocol is designed to be 3 min × 85%, 2 min × 95%, and 1 min 100% VO2max. One minute of passive recovery was given between intensities (the participants were not asked to sit on the bicycle ergometer). After completing the HIIE protocol, participants were asked to cycle actively for 3 min at an intensity of 40% VO2max. The HIIE protocol was applied again after the active recovery period. (2 × [3 min × 85%, 2 min × 95%, and 1 min 100% VO2max]). After the exercise, they were subjected to a 30% VO2max cooling phase for 3 min.

#### Moderate-intensity continuous exercise

A standard warm-up of 10 min and an active rest of 3 min were given before each exercise session. Afterward, the exercise protocol was started when the participants were ready. The MICE protocol is designed to be 21 min × 65% VO2max. After the exercise, they were subjected to a 30% VO2max cooling phase for 3 min.

### Stroop test

The Stroop test is a neuropsychological test that reflects frontal region activity. It was found that saying the names of items or colors takes longer than reading the words accompany them, and it has been demonstrated that this phenomenon is known as the “color-word interference effect” [34]. There were three sections to the Stroop task: neutral, congruent, and incongruent. To answer, participants had to use their right index and ring fingers to hit either the “←” or “→” direction button. The mistake rate and reaction time were measured. There were 4 blocks in the Stroop task: 25 neutral, 25 congruent, and 25 incongruent. The baseline was recorded for 30 s at the beginning and end of the task, and the stimulus was visible on the screen for 2000 milliseconds or until a response was produced. At intervals of 1000 ms, stimuli were delivered. Responses delivered between 200 and 2000 ms after the stimulus was shown were deemed appropriate. Answers made when the participant clicked the wrong color button or when they were above the acceptable time range (200–2000 ms) were deemed erroneous. The text was all Turkish in character. The Stroop task was designed in Psychtoolbox for MATLAB 2018. The environment where the Stroop test is applied is completely free of sound and noise. Only the expert who performed the test was present during the test. All variables in the external environment were stabilized. The validity and reliability of the Stroop test have been proven in previous studies [35]. Validity and reliability were not tested in this study.

### Blood sampling

Participants were not allowed to take drugs, caffeine, alcohol, or performance-enhancing ergogenic supplements or exercise until 48 h before the study. Venous blood samples were taken while the participants were in a sitting position. Dry tubes with gel separators (Vacuette, Greiner Bio-one GmbH, Kremsmünster, Austria) containing a clot activator were used to obtain the serum. The blood samples were centrifuged for 10 min in the Medical Biochemistry Laboratory of Atatürk University Faculty of Medicine, after which the serum was separated. After centrifugation, the serum samples were aliquoted and stored in a freezer (HERA Freeze, Thermo Fisher Scientific, Waltham, MA, USA) at -80 °C until analysis .

### Biochemical analyses

Serum BDNF, S100B and NSE levels were measured via ELISA with commercially available ELISA kits according to the manufacturers’ instructions.

### Statistical analysis

The data of all participants in the study were included in the statistical analysis. There was no missing data. The G-power sample calculation program was used to calculate the minimum number of participants required for the study (version 3.1.9.4) [36]. In this study, BDNF, S100 and NSE levels were evaluated separately for sample calculation. Since there is no study in the literature similar to the study protocol of the planned study, the minimum number of participants required for the study was calculated by the program. The program inputs were as follows: F tests (ANOVA) and type I error (α): 0.05; power of the test (1-β): 0.90, effect size: 0.40 (medium ) [36, 37]. Accordingly, the sample size was calculated to be 28. All the statistical analyses were performed with the SPSS 25 package (IBM Corp. Released 2012. IBM SPSS Statistics for Windows. Version 21.0. Armonk. NY: IBM Corp). The normality of the distributions of the data was checked with the Shapiro-Wilk test. Normally distributed data are presented as the mean ± standard deviation. One-way analysis of variance (ANOVA) with repeated measurements was used to identify differences between measurement points for all values. Compliance with the sphericity assumption was checked with Mauchly’s test. Epsilon (ε) values for degrees of freedom were examined under conditions where the sphericity assumption was not met (*p* < 0.05). Greenhouse-Geisser correction was applied for ε < 0.75 and Huyn-Feldt correction was applied for ε > 0.75. Pairwise comparisons between measurements were tested by Bonferroni post-hoc analysis. Moreover, the effect size was calculated with the partial eta squared coefficient (η_p_^2^). Accordingly η_p_^2^ values were evaluated as; 0.099 (small), 0.0588 (moderate), 0.1379 (large) effect [38]. The significance level for all analyses was accepted as *p* < 0.05.

## Results

The demographic data and characteristics of the participants are presented in Table 1. The elite athletes participating in the study were ages 24.43 ± 4.72 years, training experience 14.45 ± 5.89 years, their heights are 177 ± 7.15 cm, body mass 74.64 ± 7.82 kg, BMI 22.42 ± 5.12 kg/m^2^, fat mass 14.43 ± 6.30%, and muscle mass 86.57 ± 11.02%.

**Table 1:** Characteristics of the participants

Variables	Mean ± SD	Min-Max
Age (years)	24.43 ± 4.72	19.0–26.0
Training experience (years)	14.45 ± 5.89	8.0–17.0
Height (cm)	177 ± 7.15	172.0-184.0
Body mass (kg)	74.64 ± 7.82	57.1–83.3
BMI (kg/m^2^)	22.42 ± 5.12	17.7–28.6
Fat mass (%)	14.43 ± 6.30	5.4–22.4
Muscle mass (%)	86.57 ± 11.02	78.2–94.6

*Abbreviations*: *BMI* Body mass index, *Min-Max* Minimum-Maximum

**Fig. 2:**
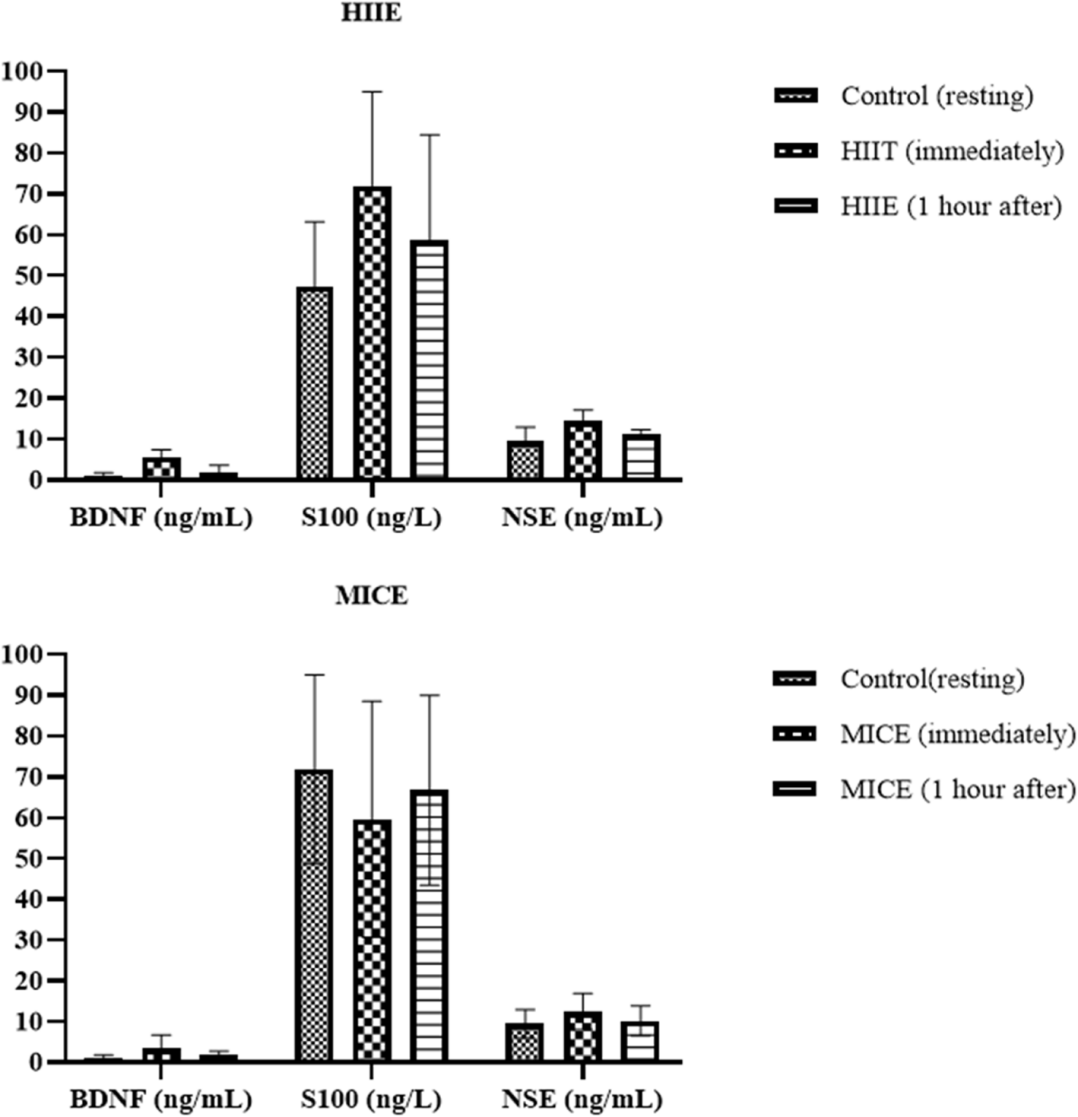
Biochemical changes according to exercise type compared to those in the control group. HIIE: high-intensity interval exercise; MICE: moderate-intensity continuous exercise; BDNF: brain-derived neurotrophic factor; S100B: S100B protein; NSE: Neuron-specific enolase

**Fig. 3:**
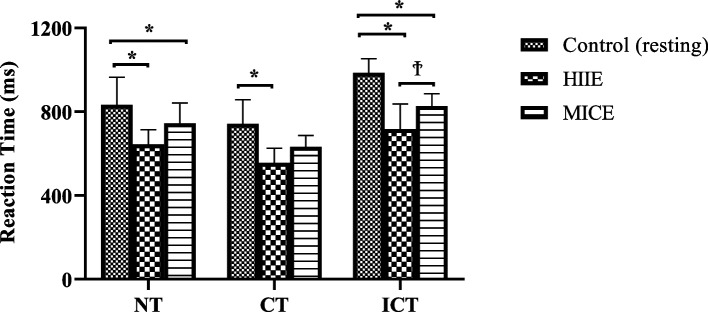
Stroop task reaction times (ms) under different supplement conditions; NT: Neutral task, CT: Congruent task, ICT: Incongruent task; *: significantly different according to CONT values (*P* < 0.05); Ϯ: significantly different according to HIIE values (*P* < 0.05)

**Fig. 4:**
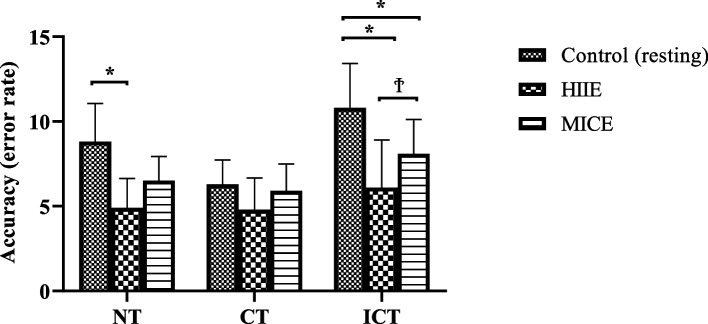
Stroop task accuracy (error rate) under different supplement conditions; NT: neutral task, CT: congruent task, ICT: incongruent task; *: significantly different according to CONT values (*P* < 0.05); Ϯ: significantly different according to HIIE values (*P* < 0.05)

 Serum BDNF, S100B and NSE levels were measured after each session and the results are presented in Table 2; Fig. 2. The results of the cognitive performance test after each session are presented in Fig. 3 (reaction time of the Stroop test) and Fig. 4 (accuracy rate of the Stroop test).

**Table 2:** Biochemical results after each session

Variables	CON (resting) Mean ± SD	HIIE (immediately) Mean ± SD	HIIE (1 h after) Mean ± SD	MICE (immediately) Mean ± SD	MICE (1 h after) Mean ± SD	F	* P *	η_p_^2^
BDNF (ng/mL)	1.24 ± 0.54	5.65 ± 1.79^a^	1.94 ± 1.65^b^	3.38 ± 1.29^c^	1.92 ± 0.79^**b**^	2.142	**< 0.001**	0.589
S100B (ng/L)	47.39 ± 15.78	71.92 ± 23.05^a^	58.57 ± 25.89	59.62 ± 28.90	55.38 ± 13.79	3.427	**< 0.001**	0.427
NSE (ng/mL)	9.51 ± 3.44	14.57 ± 2.52^a^	11.11 ± 1.17^b^	12.48 ± 4.45	10.18 ± 3.67	1.475	**< 0.001**	0.312

*Abbreviations*: *CONT* Control group, *HIIE* High-Intensity Interval Exercise, *MICE* Moderate-Intensity Continuous Exercise, *BDNF* Brain-Derived Neurotrophic Factor, *S100B* S100B protein, *NSE* Neuron-specific enolase

^a^significantly different according to CONT values (*P* < 0.05)

^b^significantly different according to HIIE (immediately) values (*P* < 0.05)

^c^significantly different according to HIIE (1 h after) values (*P* < 0.05)

Repeated measures one-way ANOVA, BDNF (ng/mL) (F = _2.142_, *P* < 0.001, η_p_^2^ = 0.589), S100B (ng/L) (F = _3.427_, *P* < 0.001, η_p_^2^ = 0.427), and NSE (ng/mL) (F = _1.475_, *P* < 0.001, η_p_^2^ = 0.312) revealed statistically significant differences in these variables (Table 2).

## Discussion

In this study, we compared the effects of HIIE and MICE on neurobiological markers and cognitive performance. The main most general findings were as follows: erum BDNF, S100B, and NSE levels were significantly different between the HIIE and groups; HIIE was superior to MICE in terms of both the accuracy rate and reaction time; and MICE also significantly influenced cognitive performance and the expression of neurobiological markers (BDNF, S100B, and NSE) according to the control conditions.

A significant difference in BDNF expression was observed immediately after HIIE compared to that in the control group. BDNF levels approached basal levels one hour after HIIE, and a significant difference was detected compared to the values measured immediately after HIIE exercise. Immediately after MICE, a significant difference was found in BDNF compared to the values recorded one hour after HIIE exercise. BDNF levels approached basal levels one hour after MICE, and a significant difference was detected compared to the values measured immediately after HIIE exercise. A significant difference in S100 was observed immediately after HIIE compared to the control values. S100 levels measured one hour after MICE were significantly different from those measured immediately after HIIE exercise. A significant difference in NSE was observed immediately after HIIE compared to the control values. NSE levels approached basal levels one hour after HIIE, and a significant difference was detected compared to the values measured immediately after HIIE exercise.

A significant difference in reaction times difference was detected in the NR task after HIIE and MICE compared to the control values. In the CR task, a significant difference was found after HIIE compared to the control values. In the ICR task, a significant difference was observed after both HIIE and MICE compared to the control values. Additionally, the results obtained after HIIE differed significantly from those after MICE. The best reaction times occurred immediately after HIIE. For the error rates, a significant difference was detected in the NR task after HIIE compared to the control values. In the ICR task, a significant difference was found after both HIIE and MICE compared to the control values. Additionally, the results obtained after HIIE differed significantly from those after MICE. The highest accuracy rate occurs immediately after the HIIE.

By changing blood flow to cerebral tissues [8], releasing BDNF [14], and activating brain regions, such as the prefrontal cortex and cingulate gyrus [3, 4], exercise has been demonstrated to enhance cognitive performance. The HIIE has emerged as a time efficient and time-consuming training method that consists of completing brief periods of intense exercise with active or passive recovery between each interval [39]. This results in greater stimulation of the cardiovascular and muscular systems. Nevertheless, the benefits of HIIE on cognition and other psychological functions are poorly understood [11]. On the other hand, MICE is a training type of lower intensity but longer duration. In this study, both HIIE and MICE increased cognitive performance compared to that in the control condition. However, HIIE induced a greater increase in cognition than MICE. Additional data are available to distinguish the effects of HIIT and MICE on inhibitory control. It was reported that both HIIT and MICE reduced response interference during a Stroop task when compared to a pre-exercise baseline assessment; however, this influence was only maintained for 30 min after HIIT, implying that acute HIIT might well have extended gains to aspects linked to executive functions compared with MICE [40]. However, to counteract the possible learning effect of repeated exposure to the Stroop task, this research lacked a non-exercise control condition. Recently, executive function in response to 20 min of acute MICE and 9 min of acute HIIT was examined via a modified flanker test [41]. The results revealed an overall decrease in reaction time following MICE and HIIT compared to that in the control (rest) condition, and only HIIT was linked to improved response accuracy when the task requirements for executive functions were increased. P3 is an event-related brain potential that it commonly measured as an index of the regulation of attention underpinning executive function after a single bout of exercise training [42]. SC Kao, DR Westfall, J Soneson, et al. [41] demonstrated that MICE enhanced P3 amplitude but that HIIT decreased P3 amplitude compared to the control condition. However, the same study [41] showed that HIIT had additional benefits on response accuracy during the flanker task. Similarly, in another study [42], although MICE was shown to have a greater P3 amplitude than HIIT, similar short-term facilitating effects on executive function following HIIT and MICE were demonstrated. HIIT and MICE may produce distinct patterns of selective attention resource allocation and have distinct impacts on information processing in support of inhibitory control. Since the current study did not perform a neuroelectric assessment ( electroencephalography (EEG)), the neural mechanisms through which HIIT increases cognitive performance more than MICE are currently unknown. However, it can be speculated that differences in executive performance caused by acute HIIE or MICE might be attributable to exercise-induced enhancement in catecholamines and subsequent locus coeruleus activation [43] which might not only enhance attention and vigilance, but also have a powerful stimulatory impact on cognitive performance dependent on the prefrontal cortex [44].

Several investigations have also focused on directly comparing the effects of HIIE and MICE on brain functions; however, these findings are not always clear and might sometimes seem to contradict one other. LT Ferris, JS Williams and C-L Shen [20] was the first to conclude that high intensity aerobic exercise had a greater influence on cognitive function than MICE. Conversely, MICE was previously shown to be more effective than HIIE in terms of enhancing cognitive performance in older adults [9]. Moreover, in another study, both HIIE and MICE were found to decrease reaction time in adolescents [45]. These controversial findings might be explained by the “3W1H” framework of Y-K Chang, KI Erickson, E Stamatakis, et al. [46] in which participants’ characteristics (training status, sex, age etc.) and timing of testing are suggested to moderate the outcomes. In this regard, the most obvious difference between these and the current study is the “training status” of the participants. Highly trained elite level athletes participated in the present study, however, the “inverted-U hypothesis” was not. In parallel, the results of S Hüttermann and D Memmert [10] showed that the inverted-U hypothesis of exercise training has an impact on cognition only in non-athletes and revealed a linear improvement in attentional performance in elite athletes up to the greatest intensity of exercise (70% of maximum heart rate). Furthermore, in sedentary individuals, S-C Kao, ES Drollette, JP Ritondale, et al. [42] suggested that the optimal stimulation of the locus coeruleus-norepinephrine (LC-NE) system was only induced by MICE, as evidenced by an increase in P3 amplitude after the flanker test, but HIIE could not generate effective activation of the LC-NE to modify the availability of attentional resources during inhibitory control operations. Finally, both moderate intensity (60% of heart rate reserve (HRR)) and high intensity (80% HRR) aerobic exercise were reported to have significant beneficial effects on reaction time during Stroop tasks in trained individuals [47]. There seems to be a physiological difference between athletes and non-athletes, as indicated by repeated observations in this topic. Specifically, there is a link between the magnitude of the beneficial effects of exercise on cognition and the “anaerobic threshold” where an exponential increase in lactate concentration is associated with a decrease in executive functions. Hence, it might be proposed that an individual’s fitness level favorably influences acute cognitive performance responses to higher intensity exercise training [48].

In the present study, elevated plasma levels of BDNF immediately after both HIIE and MICE were demonstrated compared to those in the control session. However, this trend was reversed by 60 min (Table 2). Immediately after exercise, HIIE had a greater influence on BDNF than MICE. Current results are in line with the observation that exercise induced elevation in BDNF expression is proportional to the “intensity” of exercise training [20, 49–51]. Both sprint interval training (SIT) consisting of four 30-s all-out sprints and MICE increased plasma BDNF concentrations, but SIT was more effective than MICE was [49]. A similar time course of recovery in BDNF after exercise training (30 min) was also reported in the aforementioned study [49]. Moreover, numerous additional studies have shown that HIIT elevates post-exercise BDNF concentrations more than MICE, and that these concentrations return to baseline within 30–60 min [20, 50, 51]. The restoration of BDNF to resting conditions after exercise oscillates between 30 and 60 min, which may be related to the blood analysis method used. The serum BDNF concentration exhibited a greater proportionate increase in response to exhaustive exercise than did the plasma BDNF concentration, which also returned to baseline concentrations more slowly [16]. Furthermore, the greater increase in BDNF compared to MICE with HIIT in the previous and current studies may be attributable to lactate metabolism, even though lactate levels were not measured in this study. Exercise acutely enhances the lactate concentration in brain tissue, in turn, inducing a cascade that stimulates BDNF expression via an increase in the activation of the SIRT1 and PGC1α nerve pathways [52]. This proposed mechanismof action was supported by LT Ferris, JS Williams and C-L Shen [20] and JT Reycraft, H Islam, LK Townsend, et al. [49] who reported a positive correlation between changes in the serum or plasma BDNF concentration and lactate concentrations. As a consequence, higher lactate concentrations produced by HIIT can be considered a guiding factor in activating brain BDNF expression, which may increase neuronal BDNF levels over time for release with consecutive training sessions. In contrast, it should be noted that a subgroup analysis of a meta-analysis, that included four studies [53], revealed no change in BDNF concentrations between HIIT and MICE. However, the fitness status of the participants was either sedentary or had a low level of physical activity. The physiologically distinct differences in lactate kinetics between sedentary and trained individuals may explain why the highly trained athletes in this study produced greater BDNF responses in the HIIT session. To reach firm conclusions, further research is needed to directly compare the effects of HIIT and MICE on BDNF levels in participants with both low and high physical fitness status.

S100B is known as a protein produced by brain cells and is released into the bloodstream in conditions such as brain injury or infection. Exercise increases S100B release by increasing brain cell activity and blood flow to the brain [54]. However, these increases are generally considered temporary and harmless [23]. Exercise is thought to have many beneficial effects on brain health. However, there is not enough information on what intensity of exercise is required and the long-term effects of temporary increases in S100B levels.

The beneficial effects of exercise on neurotrophic factor levels are also being increasingly investigated, and exercise is becoming a therapy for patients with neurodegenerative diseases [55]. S100B appears to be an important marker for these processes as an indicator of blood brain barrier breakdown. However, despite the abundance of published studies on S100B as a diagnostic biomarker in a clinical setting, S100B has been characterized only loosely in the context of exercise. Most studies on the relationship between serum S100B levels and exercise have shown that serum S100B levels are greater after exercise than at baseline [56–61]. However, a few studies have not shown this relationship [62–64]. Hypotheses in studies investigating this relationship show significant differences in terms of exercise effectiveness, type of exercise, and sampling methods. Studies that do not show an increase in S100B levels during exercise mostly involve lower intensity exercise (based on the absence of induced cardiovascular stress) and non-match situations involving light running and changes in direction. MICE is a less intense type of exercise than HIIE is [65]. Therefore, triggering S100B release may not be sufficient due to decreased muscle damage and stress. In addition, moderate-intensity continuous exercise typically lasts longer and requires less anaerobic capacity, which may result in less muscle damage and stress through a different energy metabolism pathway than HIIE. Therefore, a significant increase in S100B levels may not be observed or may remain at normal levels.

The current study showed a significant increase in S100 levels immediately after HIIE compared to those in the control group. However, one hour after HIIE, the levels returned to baseline. Although an increase in S100 levels immediately after MICE compared to basal levels was observed, no significant difference was detected. Again, one hour after MICE, the levels returned to baseline. The results indicate that S100B levels may increase after HIIE, possibly due to the intensity of HIIE causing damage to muscle cells and triggering S100B release. However, these increases are temporary, and S100B levels return to normal levels. Additionally, regular exercise is believed to be beneficial for overall brain health, and temporary increases in S100B levels may be an indicator of these benefits, according to the current study.

NSE is an enzyme that is considered an indicator of neuronal damage. High levels of NSE can be detected in various conditions such as neurological disorders, brain injury, trauma, infection, or tumors [66]. Exercise is a physical stress that causes various biochemical changes. The effect of exercise depends on the type, intensity, duration, and individual factors [67]. In many recent studies, exercise was shown to affect NSE levels. In particular, intense exercise (HIIE or maximal exercise) triggers neurological damage and increases NSE levels [68, 69]. However, it has been shown that low and moderate-intensity exercise (such as light running, swimming, or walking) does not affect NSE levels or have a limited effect on the activity level [57, 61, 70]. Therefore, exercise itself does not cause neuronal damage but may cause a temporary increase in NSE levels due to biochemical changes in the brain. In the present study, there was a significant difference in NSE levels immediately after HIIE compared to the control values. NSE levels approached baseline levels one hour after HIIE, and a significant decrease was detected compared to the values immediately after HIIE exercise. Although there was an increase in NSE levels after MICE, no significant differences were found compared to baseline levels. In conclusion, the effect of exercise on NSE levels can vary depending on the type, intensity, duration, and individual factors. However, it has been shown that low and moderate-intensity exercise do not trigger neuronal damage or affect NSE levels.

There are several possible reasons why low to moderate intensity exercise does not trigger neuronal damage or affect NSE levels. First, low to moderate intensity exercise is not as intense as more vigorous exercise therefore, they do not cause neuronal damage to the same extent. In these exercises, muscle cells are under less stress because they consume less oxygen to meet their energy needs and accumulate less lactic acid [71]. Second, low to moderate intensity exercises can be performed for longer periods of time, which provides a slower and less taxing energy supply to the muscles. This can prevent the occurrence of neuronal damage [72]. Finally, it is believed that low to moderate intensity exercise results in less high-frequency vibrations, which play a role in triggering neuronal damage. In vigorous exercise, muscle cells contract more quickly and forcefully, which causes high-frequency vibrations and increases the risk of neuronal damage [73]. Therefore, it is generally thought that low to moderate intensity exercise does not affect NSE levels.

This study has several limitations. First, the sample size was relatively small. Second our study population was limited to male participants and the results cannot be applied to female participants. Blood samples were drawn not only immediately after exercise but also one hour after exercise, allowing additional sample time points to better characterize the temporal kinetics of each biomarker. It also allows for accurate comparisons of previous studies with exercises performed at different intensities and varying sampling times.

In conclusion, both HIIE and MICE have favorable effects on improving cognitive performance and neuroprotection in an athlete population. HIIE is considered to be superior to MICE in improving neuroprotection (demonstrated by BDNF) and cognitive performance (demonstrated by accuracy rate and reaction time). Our study has remarkable results demonstrating the benefits of HIIT on neuroprotection and cognitive performance. HIIE is recommended instead of MICE, especially in sports types where cognitive performance is more important.

### Limitations

In this study, despite considering the circadian rhythm, specific controls were not implemented regarding the effects of physical activity or stimulant intake prior to exercise and testing procedures. Therefore, it should not be overlooked that such interactions might influence physiological markers. Additionally, the risk of carry-over effects is a significant concern when interventions are conducted following exercise protocols such as HIIE and MICE. Taking this carry-over effect risk into consideration, researchers should be mindful of this factor when designing or interpreting their studies. Careful planning may be required in selecting control and intervention groups, ensuring proper randomization, and managing the time intervals between interventions. Researchers should plan meticulously to minimize this risk and consider this effect when interpreting results. One limitation of this study is that the exercise interventions consisted of only a short series of sessions. Such a brief intervention may not be sufficient to evaluate the long-term effects of exercise and could hinder our understanding of potential dose-response relationships. Future studies are needed to assess the effects of longer or repeated exercise interventions, exploring various doses and durations more comprehensively. The results of this study may contain certain limitations stemming from the characteristics of the sample. Specifically, the fact that the participants in our study were exclusively elite athletes might constrain the generalization of the findings to the broader athlete population or a more extensive audience. The unique training, dietary, and lifestyle habits of elite athletes could raise questions about how these findings can be applied to the broader athlete populace. Consequently, these results might need validation or confirmation in a more extensive athlete sample. Moreover, the size and diversity of the sample could influence the overall validity of the study, which should be addressed in future research through more comprehensive sample selection.

## Data Availability

The datasets used and/or analyses during the current study are available from the corresponding author on reasonable request.
